# When Shepherds Shed: Trajectories of Weight-Related Behaviors in a Holistic Health Intervention Tailored for US Christian Clergy

**DOI:** 10.1007/s10943-023-01910-8

**Published:** 2023-09-14

**Authors:** Jia Yao, Dori Steinberg, Elizabeth L. Turner, Grace Y. Cai, Jacqueline R. Cameron, Celia F. Hybels, David E. Eagle, Glen Milstein, Joshua A. Rash, Rae Jean Proeschold-Bell

**Affiliations:** 1 Global Health Institute and Center for Health Policy & Inequalities Research, Duke University, Durham, NC USA; 2 School of Nursing and Global Health Institute, Duke University, Durham, NC USA; 3 Department of Biostatistics and Bioinformatics and Global Health Institute, Duke University, Durham, NC USA; 4 Trinity College of Arts and Sciences, Duke University, Durham, NC USA; 5 Department of Internal Medicine, Section of Palliative Medicine, Department of Preventive Medicine, Department of Religion, Health and Human Values, Rush University, Chicago, IL USA; 6 Department of Psychiatry and Behavioral Sciences, Center for the Study of Aging and Human Development, Duke University Medical Center, Durham, NC USA; 7 Department of Psychology, The City College of New York, New York, NY USA; 8 Department of Psychology, Memorial University of Newfoundland, St. John’s, NL Canada

**Keywords:** Occupational health, Obesity, Clergy, Health behavior, Tailor, USA

## Abstract

Maintaining healthy behaviors is challenging. Based upon previous reports that in North Carolina (NC), USA, overweight/obese clergy lost weight during a two-year religiously tailored health intervention, we described trajectories of diet, physical activity, and sleep. We investigated whether behavior changes were associated with weight and use of health-promoting theological messages. Improvements were observed in sleep, calorie-dense food intake, and physical activity, with the latter two associated with weight loss. While theological messages were well-retained, their relationship with behaviors depended on the specific message, behavior, and timing. Findings offer insights into weight loss mechanisms, including the role of theological messages in religiously tailored health interventions.

## Introduction

The prevalence of obesity among United States (US) adults is 42.5% and has been increasing over the past two decades (Centers for Disease Control and Prevention [CDC], 2020a). Across the occupations in the US, high obesity rates have been reported among clergy. In two studies with adjusted comparisons that account for education, employment, and insurance status, United Methodist clergy exhibited an obesity prevalence 10.3 percentage points higher than the general population in North Carolina (NC) (39.7% vs. 29.4%), and 10.8 percentage points higher in Kansas (40.4% vs. 29.6%) (Lindholm et al., 2016; Proeschold-Bell & LeGrand, 2010). Above-average obesity rates have also been found across the US for Evangelical Lutheran Church in America clergy (Halaas, 2002), African Methodist Episcopal clergy (Baruth et al., 2014), and Wesleyan clergy (Mook, 2019). The reasons for these high rates of obesity in clergy is not entirely known. However, clergy face multiple challenges to exercise and healthy eating, including (1) being in a sedentary vocation, (2) frequently spending evenings away from home for meetings and hospital visits, (3) high time demands, and (4) little schedule predictability (Proeschold-Bell & Byassee, 2018). Obesity both causes and complicates a number of chronic diseases, including diabetes, arthritis, asthma, joint disease, heart disease, and hypertension (National Heart, Lung, and Blood Institute [NHLBI], 1998; Reynolds & Mcllvane, 2009). It is therefore clinically important to identify ways to address the high rates of obesity in clergy.

As weight loss can be difficult to achieve, international guidelines for the management of overweight and obesity for adults in primary care recommend comprehensive lifestyle changes that include changes in diet and physical activity (Semlitsch et al., 2019). Researchers have reviewed studies to determine the relative benefit of diet versus physical activity. Diet-only programs are overall as effective as combined diet and physical activity programs in the short term and less effective in the long term, while physical activity-only programs are less effective than combined programs in the short and long term (Johns et al., 2014).

There is increasing awareness of the role of sleep in weight regulation. A meta-analysis of observational studies reporting on more than 5 million participants concluded that short sleep duration was associated with a 1.38-fold increase in risk of obesity, though significant between-study heterogeneity was present (Itani et al., 2017). Moreover, intervention studies indicate that change in sleep is associated with weight loss and management. In a study of overweight and obese adults participating in a caloric restriction intervention, researchers found a positive relationship between sleep duration and loss of body fat; they also found that better sleep quality was associated with greater loss of fat mass (Chaput & Tremblay, 2012).

Although Americans recognize that changing one’s behaviors is key to weight loss, there are unanswered questions about how to both motivate changes and sustain motivation over time. Tailoring behavior change programs to connect with one’s deeply held beliefs may help motivate behavior change and sustain the motivation. An example would be to connect weight loss messages with theological beliefs among clergy. Weight loss messages tailored to one’s faith could be welcomed by clergy, as found in a study of seminary students in which most expressed a desire for health to be taught and emphasized in seminary training (Bopp & Baruth, 2017).

A holistic health intervention, named Spirited Life, invited all United Methodist clergy of any weight from NC to participate (Proeschold-Bell et al., 2013). At enrollment, 49% of participants were obese and 34% were overweight (Proeschold-Bell et al., 2017). The intervention was tailored for clergy and incorporated Christian faith-based messages that highlight the theological importance of health and health behaviors. The intervention encouraged each participant to set their own holistic health goals, which for many included weight loss. Selected components of the intervention guided participants on healthy eating and physical activity. The two-year intervention effect on weight in participants who were obese at enrollment was an average of 1.8 kg weight loss per person (95% CI [0.0 kg, 3.6 kg]); in participants who were overweight or obese at enrollment, the two-year intervention effect was 2.6 kg weight loss on average (95% CI [1.4 kg, 3.8 kg]). This level of weight loss is modest, and consistent with other trials conducted in real-world settings (Dunkley et al., 2014). In addition, participants with high body mass index (BMI ≥ 40) pre-intervention sustained their weight loss for 18 months after the two-year intervention ended (Proeschold-Bell et al., 2020).

This paper builds on the earlier reports of Spirited Life outcomes and contributes by investigating possible mechanisms behind the weight change that participants experienced from the intervention. In this paper, the Spirited Life trial data are used to describe the concurrent trajectory of change in weight and relevant health behaviors, including diet, physical activity, and sleep. We seek to answer 3 research questions: (1) How did health behaviors change during the intervention period? (2) Were changes in health behaviors related to weight loss? and (3) Were changes in health behaviors related to acting on the theological messages intended to promote health behaviors?

## Methods

### Intervention

The Spirited Life intervention study has been registered at www.clinicaltrials.gov (NCT01564719) and described in detail (Proeschold-Bell et al., 2013). In 2010, 1114 clergy from the NC and the Western NC Annual Conferences of the United Methodist Church enrolled in the study and were randomly assigned into one immediate intervention cohort and two waitlist cohorts. All cohorts received the holistic health intervention for 2 years in their respective timelines. The intervention consisted of multiple components, of which 4 pertained to weight loss: (1) a required workshop at the intervention’s baseline time point, plus optional workshops at the midpoint and end; (2) an online video-based curriculum named Naturally Slim^®^; (3) health coaching; and (4) small grants ($500 each) offered at the midpoint to help achieve individual health goals. Components 2–4 were optional, which imitated real-life settings.

Four theological messages tailored for Christian Methodist clergy and intended to motivate health behavior changes were conveyed through three workshops. These messages were: 1) through Incarnation, God declared human bodies as good and worthy of care; (2) willingness to accept help is important in all areas of life; (3) receiving grace for oneself (i.e., self-compassion) is important; and (4) John Wesley, the founder of Methodism, conceptualized health to include diet and physical activity.

The Naturally Slim^®^ curriculum was offered to participants within the first 4–5 months of the intervention and was engaged by most participants. It consisted of 10 weekly one-hour videos, encouraging eating only when hungry, eating smaller portions, reducing sugar intake, and walking daily. Notably, it did not promote eating nutrient-dense foods more often.

Health coaches were drawn from a range of careers and trained by a psychologist certified in motivational interviewing and health coaching techniques. Each participant was assigned to a health coach, and if interested, participated in individual coaching over the phone once a month for up to 23 months. Sessions focused on goal setting (weight-related or not) and habit formation. The health coaches drew upon the 4 health-promoting theological messages when working with the participants.

During the intervention period, participants were invited to health assessments at baseline and three follow-up time points (12, 18, and 24 months). Metabolic syndrome risk factors, including weight, were measured at each assessment. At the time of the assessment, participants were informed whether their numbers were within healthy ranges.

All study procedures were approved by the Duke University Institutional Review Board. All study participants gave informed consent.

### Analysis Sample

This paper consists of secondary analyses of data from the Spirited Life study, in which participants were randomly assigned to start the intervention immediately, with a one-year delay, or with a two-year delay. For this paper, the baseline time point is defined as immediately before the participant started receiving the intervention (i.e., we report outcomes based on receiving the intervention, even in the few cases where random assignment was broken). Survey and health assessment data, collected at 4 time points throughout the intervention, were used for analyses.

Only Spirited Life participants who were overweight or obese at baseline were eligible for this paper’s analyses. To be included, such participants must have provided data at baseline plus at least one follow-up time point. Also, for at least one of the 4 study time points, the participant had to provide data for all 4 key analysis variables: weight, diet, whether their amount of physical activity met the minimal requirement for health, and sleep quality. Data were excluded in cases where a female participant was pregnant or within 6 months postpartum at an assessment time point.

### Measures

Weight and height were measured by study staff and used to calculate body mass index (BMI) using the National Heart, Lung, and Blood Institute definition (NHLBI, 1998). The diet measure drew on items from the 2009 Behavioral Risk Factor Surveillance System (CDC, 2020b) and the National Health and Nutrition Examination Survey Food Frequency Questionnaire (CDC, 2020c). Participants reported how many times per day, week, or month they consumed 5 kinds of calorie-dense food: (1) fries and chips, (2) sugared drinks, (3) sweets, (4) fast food, and (5) non-whole grain breads, as well as 4 kinds of nutrient-dense food: (1) whole grain breads, (2) fruits, (3) green salads, and (4) vegetables. We calculated number of times consumed per month for each kind of food.

In order to summarize data and reduce the number of variables on diet, principal-component factor analysis was conducted on the 9 food intake frequencies at baseline. Two principal components were identified and standardized so that each component had a mean of 0 and a standard deviation (*SD*) of 1 at baseline. Because one component yielded high loadings on the 5 kinds of calorie-dense foods, and the other component high loadings on the 4 kinds of foods that are nutrient-dense, we named them respectively, after standardization, as the “calorie-dense food intake score” and the “nutrient-dense food intake score”.

Physical activity was measured using items from the 2007 BRFSS questionnaire (CDC, 2020b). Two categories of activities were captured: moderate activities, which cause some increases in breathing or heart rate, and vigorous activities, which cause large increases in breathing or heart rate. Participants reported the number of days per week they did each category of activities and the amount of time per day they spent doing them, and we calculated the number of minutes per week for each category.

The World Health Organization (WHO) recommends that healthy adults do at least 150 min of moderate-intensity aerobic physical activity, or at least 75 min of vigorous-intensity aerobic physical activity, or an equivalent combination of moderate- and vigorous-intensity activity, throughout the week (WHO, 2010). Each minute of vigorous activity is treated as being equal to two minutes of moderate activity in calculating the minimal physical activity amount for health. In this analysis, we calculated the weighted total weekly amount of physical activity using the same formula, then dichotomized at 150 + minutes/week to generate a binary variable indicating whether the participant met the minimal physical activity requirement for health (*Yes* = 1/*No* = 0).

A single survey item measured sleep duration on a typical night. For sleep quality, participants reported whether or not they usually woke up feeling rested.

Participants were asked to check any of the 4 health-promoting theological messages taught throughout the intervention that they “drew upon to support (themselves) while changing (their) health behaviors.” These questions were included on only one survey wave whose timing varied by cohort: at 12 months post-baseline for the two-year delayed intervention cohort, and at 24 months post-baseline for the one-year delayed intervention cohort. The immediate intervention cohort was not asked these questions during the intervention period and therefore did not provide data for these analyses.

### Analyses

We described the participants’ characteristics at baseline, including demographics, weight, BMI, diet, physical activity, and sleep. For the binary and categorical variables, we reported percentages and counts; for the continuous variables, we reported means and standard deviations.

We illustrated the trajectory of weight over the course of the intervention using a figure showing means and standard errors of weight at each time point. Likewise, we illustrated trajectories of diet, physical activity, and sleep quality over time. Regression modeling of continuous outcomes over time was within the fixed effects regression framework in order to account for the repeated outcome measurements on the same individual and to control for participants’ characteristics that remained constant over time. Binary outcomes were analyzed using the modified Poisson regression (Zou & Donner, 2013). To account for potential heteroskedasticity, robust standard errors were used.

Some data were missing due to attrition from the study or missing variables from a specific time point. To assess sensitivity to missing outcome data, we created a trajectory for participants with data at all four time points for each behavioral variable (i.e., with no missing outcome data) and compared it to a trajectory for all participants, including those with data at only two or three time points.

The relationship between weight in kilograms and each health behavior over time was modeled using a multivariable linear fixed effects regression model. The health behavior measures included in the model were: the standardized principal components of diet, whether the participant met the minimal physical activity requirement for health, and whether the participant woke up rested. Additionally, the same model was fit again with weight in percentages of baseline weight and BMI as respective outcomes.

By cohort, we report means, standard deviations, percentages, and counts for the theological messages that participants indicated drawing upon to support themselves in changing their health behaviors.

Associations were estimated between levels of change in health behaviors over time and participant’s use of theological messages for health behavior change, adjusting for the baseline level of each health behavior and time in intervention. Standardized principal components of diet were each fit into linear random effects models as the outcome; whether the participant met the minimal physical activity requirement for health and whether the participant woke up rested were fit into modified Poisson models. Because 20 models were run in this set of analysis, a Bonferroni correction was performed, with which the statistical significance of each model was judged by a corrected *p* value of 0.05/20 = 0.0012.

All statistical analyses were performed using Stata version 16.1 (StataCorp, College Station, TX, 2009, RRID:SCR_012763).

## Results

This analysis sample included 777 participants at baseline. At 12 months, 96% of the participants were included in analyses; 18 months, 92%; and 24 months, 86%. Participants were predominantly male (72.1%; *n* = 560). At baseline, the average participant was 53 years old (*SD* 10.0), weighed 98.8 kg, and reported doing moderate and vigorous physical activities combined for 4.7 h (*SD* 4.4) per week. See Table 1 for a full description of the weight and health behaviors of the participants at baseline.

**Table 1:** Participants’ demographics, weight, and health behaviors at baseline (*N* = 777)

Characteristics	M (*SD*) or % (*n*)
Age in years	52.9 (10.0)
Female	27.9% (217)
Married	89.3% (693)
Weight in kilograms	98.8 (21.4)
Body Mass Index (BMI)	32.8 (6.6)
Obese (BMI ≥ 30)	57.8% (449)
Food intake in times per month^1^	
Fries and chips	10.1 (13.9)
Sugared drinks	9.0 (21.1)
Sweets	15.2 (20.0)
Fast food	8.1 (13.6)
Non-whole grain breads	9.1 (17.8)
Whole grain breads	27.6 (27.2)
Fruits	28.9 (25.9)
Green salad	16.0 (16.7)
Vegetables	30.3 (25.3)
Met minimal physical activity requirement for health^2^	66.1% (508)
Woke up feeling rested	63.7% (495)
Cohorts by intervention timing	
Cohort 1: Immediate intervention	37.8% (294)
Cohort 2: One-year waitlist	25.9% (201)
Cohort 3: Two-year waitlist	36.3% (282)

^1^Missing values varied by food intake category, with 3 being the highest number of values. ^2^Eight participants missed values for whether they met the physical activity requirement

**Fig. 1:**
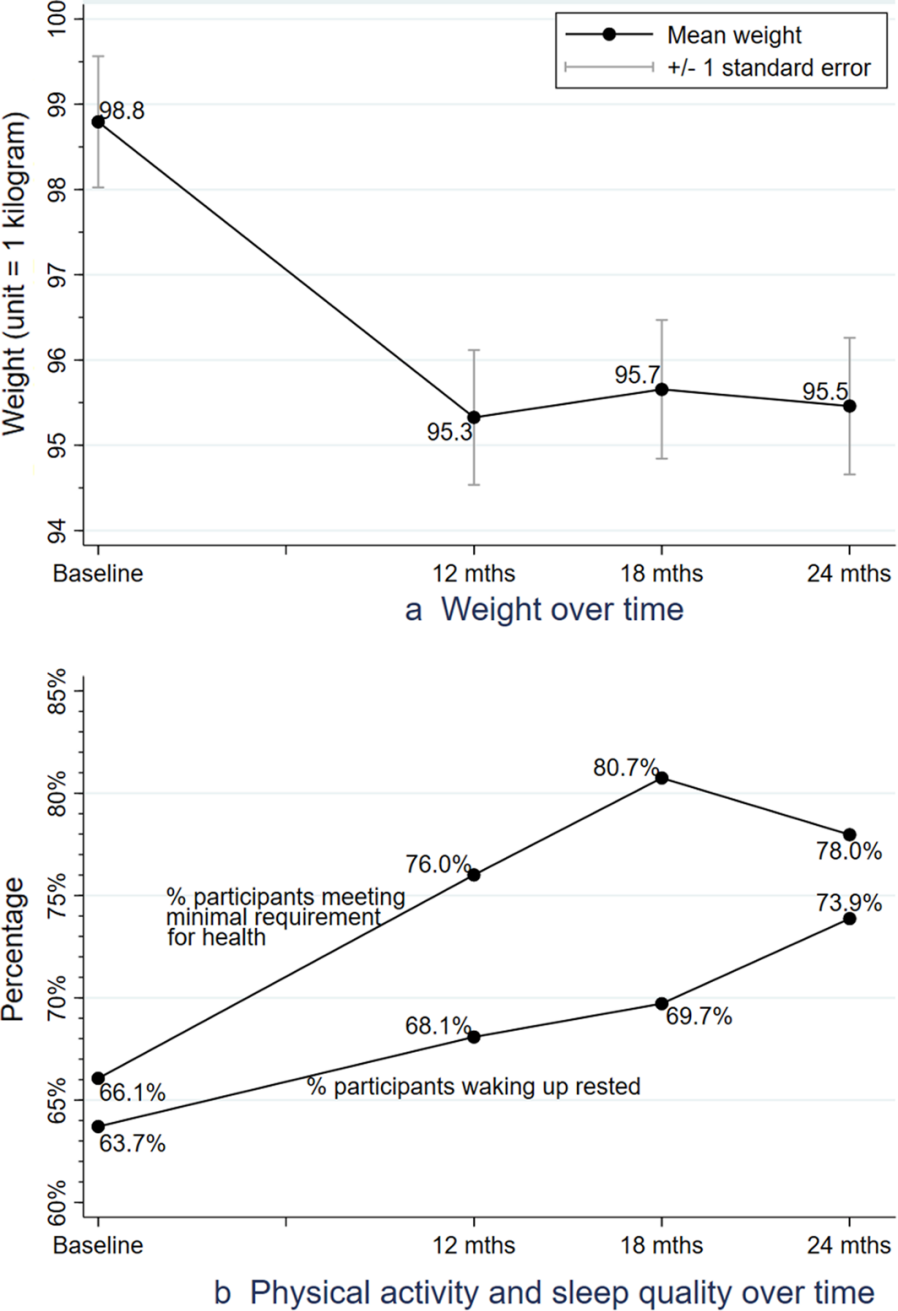
Trajectories of weight, physical activity, and sleep quality over time

Weight loss during the Spirited Life intervention is shown in Fig. 1. Participants’ average weight decreased from 98.8 kg (*SD* 21.4) at baseline and decreased to 95.5 kg (*SD* 20.6) over the two-year intervention period. The vast majority of weight loss occurred during the first year, while most participants experienced small fluctuations in weight during the second year.

**Fig. 2:**
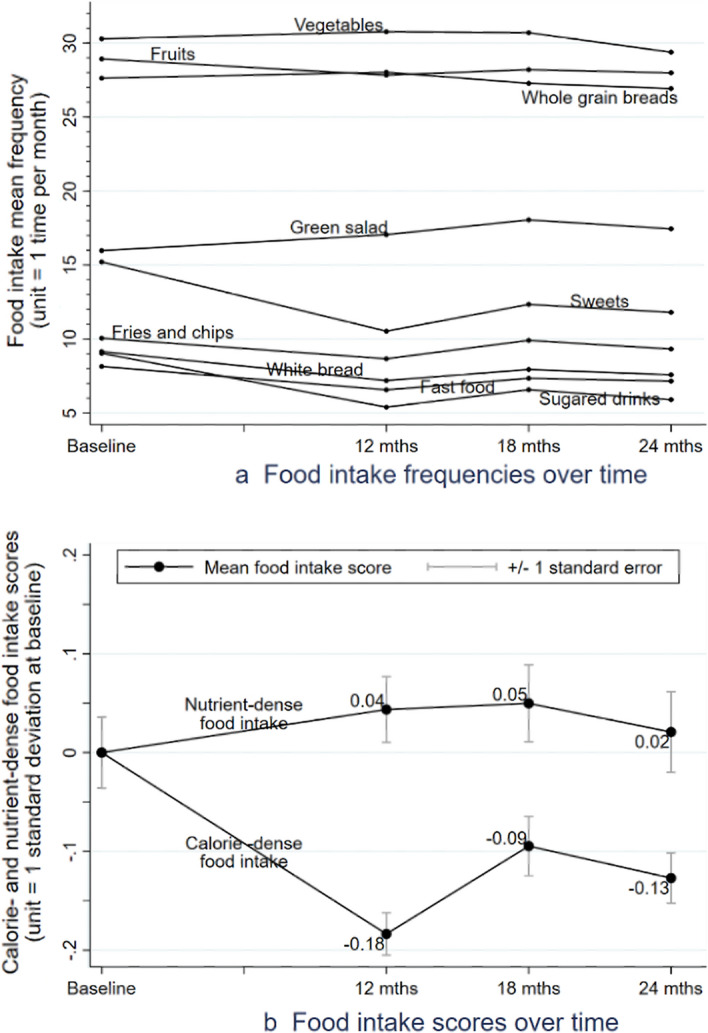
Trajectories of diet over time

Participants reported high frequencies of calorie-dense food intake at baseline (Table 1). During the past 30 days, they ate fast food an average of 8 times and sweets an average of 15 times. Participants’ calorie-dense food intake decreased during the intervention, with some fluctuations within the second year (Fig. 2). Between baseline and 12 months, participants reduced their intake of sweets and sugared drinks (mean decrease 5 times/month for each). Between 12 and 18 months, intake increased for each type of calorie-dense food, although the increases were only statistically significant for fries and chips (*p* = 0.027) and sweets (*p* = 0.011). Significant decreases between baseline and 24 months were found for sweets (*p* < 0.001), sugared drinks (*p* = 0.001), and non-whole grain breads (*p* = 0.018).

Trajectories of nutrient-dense food intake were mostly flat, with mean frequency changes from baseline within ± 2 times (Fig. 2). Green salad intake picked up over time (mean increases 1 time/month at 12 and 24 months; 2 times/month at 18 months) but did not reach statistical significance at 24 months.

The trajectory of calorie-dense food intake scores over time (Fig. 2, Panel 2b) followed a similar pattern to that of weight. Overall, the scores significantly decreased (*M* = − 0.13; *p* = 0.003), with a large decrease occurring between baseline and 12 months (*M* = 0.18). The nutrient-dense food intake scores remained stable over time and not statistically significantly different from the baseline level.

As shown in Fig. 1, the pattern of change in physical activity occurred in a direction favorable to the participant’s health. The percentage of participants meeting the minimal physical activity requirement for health significantly increased from two-thirds (66%) at baseline to more than three quarters (78%) at 24 months (*p* < 0.001), with a large increase having occurred at 18 months (81%).

Across all time points, the mean sleep duration remained 6.9 h per night (*SD* ranged 0.9–1.0). Sleep quality, however, significantly improved at each follow-up time point, from 64% well-rested at baseline to 74% at 24 months (*p* < 0.001). Unlike weight change, more than half of the percentage point increase took place within the second year of the intervention period.

In missing data analysis, we observed similar patterns when we compared the trajectories for those with data at all 4 time points to those with all available data.

The fixed effects models of weight in kilograms, weight in percentage of baseline weight, and BMI show that all three outcomes were significantly associated with calorie-dense food intake scores, nutrient-dense food intake scores, and meeting the minimal physical activity requirement, but not sleep quality (Table 2). An increase in the calorie-dense food intake score by 1 standard deviation was associated with an increase of 1.0 kg, 95% CI [0.3, 1.7] in weight. An increase in the nutrient-dense food intake score by 1 standard deviation was associated with a weight decrease of 0.6 kg, 95% CI [0.1, 1.0]. A change from meeting the minimal physical activity requirement of health to not meeting the requirement was associated with a weight increase of 0.9 kg, 95% CI [0.3, 1.6].

**Table 2:** Fixed effects models of weight and BMI with health behaviors over time

	Weight in kilograms	Percentage of baseline weight	BMI
Calorie-dense food intake score (unit = 1 *SD* at BL)	0.992 [0.329, 1.654]; *p* = .003	0.932 [0.296, 1.568]; *p* = .004	0.310 [0.106, 0.514]; *p* = .003
Nutrient-dense food intake score (unit = 1 *SD* at BL)	− 0.562 [− 1.001, − 0.123]; *p* = .012	− 0.510 [− 0.934, − 0.086]; *p* = .018	− 0.147 [− 0.290, − 0.004]; *p* = .045
Participant did NOT meet minimal physical activity requirement for health	0.928 [0.297, 1.558]; *p* = .004	0.886 [0.274, 1.498]; *p* = .005	0.364 [0.147, 0.581]; *p* < .001
Participant woke up feeling NOT rested	0.400 [− 0.248, 1.049]; *p* = .226	0.441 [− 0.201, 1.084]; *p* = .178	0.139 [− 0.083, 0.362]; *p* = .220

A total of 2642 observations from 777 participants and 4 time points in intervention was included in each model. Each model adjusted for time in intervention and specified robust standard errors. Meeting physical activity requirement for health had a prevalence of over 0.5 and therefore was reverse-coded for modeling

As shown in Table 3, the intervention’s 4 health-promoting theological messages were well-received by participants. At the end of the two-year intervention, the average participant in Cohort 2 drew upon two messages to support their health behavior changes. Receiving grace for oneself was the most drawn-upon (68%) message. Willingness to accept gifts was the least drawn-upon (33%).

**Table 3:** Utilization of health-promoting theological messages

Theological messages	Participant drew upon the message to support themselves when changing health behaviors	
	At 12 mths in Cohort 3 (*N* = 263)	At 24 mths in Cohort 2 (*N* = 168)
Number of messages, mean (*SD*)	1.9 (1.3)	1.9 (1.4)
Individual messages, % (*n*)		
In taking on human flesh, God declared our bodies good, honorable, and worthy of care. Taking care of our bodies is an appropriate response to Incarnation	52.5% (136)	54.2% (91)
Being willing to accept gifts, as well as give them, is important	32.5% (83)	32.9% (52)
Self-compassion, or receiving grace for oneself, is important	65.0% (171)	67.7% (107)
Wesley’s conception of health and wholeness includes habits of diet and exercise	39.8% (103)	41.1% (69)

As shown in Table 4, drawing upon the theological message on Wesley’s conception of health at 12 months (but not at 24 months) was significantly associated with a larger increase in nutrient-dense food intake scores across the two years. Compared to not using it, drawing upon the message was associated with a higher score of nutrient-dense food intake by 0.26 unit (95% CI [0.11, 0.41], *p* = 0.0007), which translates to 0.26 standard deviation of the intake scores at baseline. Drawing upon theological messages at 12 months was not found significantly associated with changes in calorie-dense food intake scores, meeting the physical activity requirement, or waking up rested.

**Table 4:** Associations of change in health behaviors over 24 months with drawing upon theological messages at 12 months

	Random Effects models, coefficient [95% CI]; *p*		Modified Poisson models, prevalence ratio [95% CI]; *p*	
Messages drawn upon by participant to support themselves while changing health behaviors	Calorie-dense food intake score	Nutrient-dense food intake score	Did NOT meet minimal physical activity requirement for health	Woke up NOT rested
Number of messages	− 0.03 [− 0.08, 0.02]; *p* = .2457	0.11 [0.04, 0.18]; *p* = .0015	0.87 [0.73, 1.04]; *p* = .1166	0.95 [0.85, 1.05]; *p* = .2979
*Individual messages*				
Incarnation	− 0.03 [− 0.16, 0.11]; *p* = .6906	0.21 [0.05, 0.37]; *p* = .0080	0.67 [0.44, 1.02]; *p* = .0591	0.91 [0.69, 1.19]; *p* = .4745
Accepting gifts	− 0.06 [− 0.20, 0.08]; *p* = .3925	0.20 [0.01, 0.39]; *p* = .0366	Model didn't converge	0.87 [0.64, 1.19]; *p* = .3911
Receiving grace for self	− 0.02 [− 0.15, 0.12]; *p* = .8088	0.10 [− 0.09, 0.29]; *p* = .2859	1.02 [0.64, 1.63]; *p* = .9377	0.95 [0.72, 1.25]; *p* = .7257
Wesley’s health conception	− 0.07 [− 0.19, 0.04]; *p* = .1881	0.26 [0.11, 0.41]; *p* = .0007	0.58 [0.37, 0.91]; *p* = .0176	0.90 [0.67, 1.21]; *p* = .4820

Each health behavior at follow-up time point was modeled with the number of theological messages drawn upon, or whether an individual message was drawn upon, at 12 months, adjusting for the baseline level of this health behavior and time in intervention. Meeting the physical activity requirement for health and waking up feeling rested both had a prevalence over 0.5 and therefore were reverse-coded for modeling. Each model specified robust standard errors. Due to multiple testing, Bonferroni-corrected criterion *p* = .0012 is used to determine statistical significance

## Discussion

With the obesity epidemic in the USA, motivating people to engage in and sustain healthy behaviors is paramount and challenging. Mainline Protestant clergy have high rates of obesity, and the 2-year Spirited Life intervention for clergy evidenced sustained weight loss outcomes. In this paper, we conducted secondary analyses of the Spirited Life trial data to determine what health behavior changes clergy made, whether they related to weight loss, and whether the intervention’s use of theological messages related to change in health behaviors.

We studied a sample of Methodist clergy who were obese or overweight at baseline and observed them for two years as they went through the intervention. We found that overall, the participants decreased their calorie-dense food intake, increased their physical activity, and experienced improved sleep quality. The majority of improvement occurred during the first year of intervention, corresponding with when the intervention offered the Naturally Slim® curriculum. Both a reduction in calorie-dense food intake and an increase in physical activity related to weight loss. Although not all participants increased intake of nutrient-dense food, for those who did, such an increase related to weight loss. Among the four health-promoting theological messages delivered through the intervention, one-third to two-thirds of the participants reported drawing on specific messages, especially the message on receiving grace, to motivate themselves when changing behaviors. The denomination’s founder’s message to include diet and physical activity, when conceptualizing health, related to significant increases in nutrient-dense food intake.

This paper adds to the literature through exploring the underlying mechanisms of weight loss, including reporting intermittent weight changes, changes in health behaviors over time, and participants’ utilization of health-promoting theological messages. The intermittent weight loss progress in Spirited Life participants is comparable to that of participants in other studies. In their systematic review of randomized controlled trials, Borek et al. (2018) found that the average weight loss for overweight or obese participants in group-based diet and/or physical activity weight-loss interventions was 3.49 kg, 3.44 kg, and 2.56 kg at 6, 12, and 24 months, respectively. Unlike in Spirited Life, those participants were of mixed occupations. The weight loss mechanisms that we observed are comparable to a systematic review, which found that weight loss intervention effectiveness related to self-monitoring and social support (Greaves et al., 2011).

We observed that most of the positive behavior changes in diet, physical activity, and sleep happened during the first year of the two-year intervention, corresponding to the timing of weight change. During the second year, only sleep continued in the same trends as the first year. We observed a setback in weight and all calorie-dense food intake frequencies between 12 and 18 months, which then improved again by 24 months. The fluctuations could have been related to the timing of the metabolic health assessment and feedback that participants received at baseline, 12 months, 18 months, and 24 months. One of the most widely used conceptual frameworks in health behavior research is the health belief model (HBM), which could be used to explain these fluctuations (Rosenstock, 1974). According to the HBM, an individual is likely to act when they: (1) perceive themselves as susceptible to a condition; (2) believe the condition has potentially serious consequences; (3) think that a course of action available to them will reduce the risk or potential consequences of a condition; and (4) perceive that anticipated benefits outweigh barriers. Adopting this view, participants may have been motivated and equipped to make health behavior changes during the first year when the perceived susceptibility and consequences of obesity-related morbidity were the greatest. At 12 months, they learned that their weight and metabolic health had improved; this knowledge could reduce perceived susceptibility and consequences of obesity-related morbidity and may have resulted in waning motivation to restrain from calorie-dense food intake, leading participants to loosen up on their diet. Learning that their health had worsened at 18 months would likely increase perceived susceptibility and consequences of obesity-related morbidity, resulting in a return to adopting healthy behaviors. This speculation highlights the need to maintain motivation, and the potential benefits of frequently monitoring and giving feedback on health indicators. The intervention might have been strengthened if participants had received their biometric information at just 6 months after starting the intervention, rather than waiting 12 months.

During the second year of the intervention, despite the intermittent fluctuations, improvements observed in calorie-dense food intake, physical activity, and weight were maintained at overall healthier levels compared to baseline. This observation is consistent with published results on weight loss maintenance and indicate promising long-term outcomes for the intervention (Proeschold-Bell et al., 2020).

The intervention did not focus on changing participants’ sleep behavior. However, participants experienced steady improvements in sleep quality for two years. Intriguingly, change in sleep quality did not relate to weight loss when controlling for diet and physical activity. It is possible that weight loss and sleep quality improvement are related through changes in concomitant health behaviors. A recent systematic review of 29 studies reported a robust association between better sleep quality and healthier diet (Godos et al., 2021). Similarly, improvement in physical activity is associated with improvement in sleep quality (Kelley & Kelley, 2017). Future studies are needed to tease apart the relationships between sleep, weight loss, and health behaviors.

Among the 4 health-promoting Christian theological messages delivered through the intervention, it is interesting that the one message specific to the studied denomination and its founder may have been the most motivating of behavior changes and that the message related to significant increases of food that is nutrient-dense. Participants may have been highly motivated to follow a role model that is particularly relevant in their religious culture. This finding speaks to the value of creating a faith-based intervention that is specifically tailored to its target group. This study contributes to the understanding of religiously tailored health interventions in the current trend of advancing interventions from faith-placed (i.e., the intervention takes place in religious settings) to authentically faith-based (i.e., significant religious components are integrated into the intervention) (Lasater et al., 1997; Padela et al., 2018). Going down this path, the intervention might be further strengthened by (1) embedding more specific cultural and denominational messages in the program, in contrast to general Christian messages, and (2) applying this theological messaging more broadly across components of the program, for example, adapting the Naturally Slim@ program, which is secular and speaks to the general population.

This study has several limitations. Health behaviors were self-reported, which is subject to recall and social desirability biases. In addition, we only asked about the theological messages on one survey wave, with different timing for different participant cohorts, and therefore cannot describe participants’ uptake of the messages as they progressed through the intervention. Among the four theological messages, only one (on Incarnation) is exclusively religious; the other three can be understood from both religious and secular perspectives. Although all four messages were developed in the Methodist cultural language and preached to participants in sermons at the workshops, further confirmatory research is needed to tease apart the pathways through which three messages motivate the participants toward healthy behaviors. Our sample was exclusively United Methodist clergy in NC, which limits the generalizability of our findings, especially beyond mainline Protestant clergy in the US (Holleman & Eagle, 2023).

This study also has several strengths, including its large sample size, which can be difficult to achieve in intervention studies, and longitudinal design spanning 2 years. Whereas many weight loss studies have predominantly female participants, the Spirited Life sample was over 70% male, adding to our understanding of mechanisms for weight loss among men. Also, not all weight loss intervention studies report data from the study’s midpoints, but in this paper, we use midpoint data and a total of 4 time points to indicate trajectories of weight and health behaviors over time.

## Conclusion

Clergy experience high prevalence of obesity. It is important to dissect multicomponent behavioral interventions to better understand if and when weight loss occurred, and how such weight loss was achieved. A multicomponent behavior intervention led clergy to enact healthy behavior changes that were sustained for 24 months and related to sustained weight loss. Researchers and congregations interested in developing weight loss programs for clergy may want to incorporate theological messages and fine-tune the messages to the clergy’s specific denomination and culture, in order to both motivate healthy behavior change and sustain such motivation. We also suggest frequently monitoring and providing feedback on participants’ weight and health indicators, to motivate sustained health behavior change.

## Data Availability

Participant data are confidential. Statistical analysis codes and log files are available to the journal editor and reviewers by request.
